# Role of the olivo-cerebellar complex in motor learning and control

**DOI:** 10.3389/fncir.2013.00094

**Published:** 2013-05-28

**Authors:** Nicolas Schweighofer, Eric J. Lang, Mitsuo Kawato

**Affiliations:** ^1^Division of Biokinesiology and Physical Therapy, University of Southern CaliforniaLos Angeles, CA, USA; ^2^Movement to Health Laboratory, Montpellier-1 UniversityMontpellier, France; ^3^Department of Physiology and Neuroscience, New York UniversityNew York, NY, USA; ^4^Brain Information Communication Research Laboratory Group, Advanced Telecommuniction Research Institue InternationalKyoto, Japan

**Keywords:** cerebellum, motor learning, inferior olive, electrical coupling, Purkinje cells, deep cerebellar nucleus, complex spikes, synchrony

## Abstract

How is the cerebellum capable of efficient motor learning and control despite very low firing of the inferior olive (IO) inputs, which are postulated to carry errors needed for learning and contribute to on-line motor control? IO neurons form the largest electrically coupled network in the adult human brain. Here, we discuss how intermediate coupling strengths can lead to chaotic resonance and increase information transmission of the error signal despite the very low IO firing rate. This increased information transmission can then lead to more efficient learning than with weak or strong coupling. In addition, we argue that a dynamic modulation of IO electrical coupling via the Purkinje cell-deep cerebellar neurons – IO triangle could speed up learning and improve on-line control. Initially strong coupling would allow transmission of large errors to multiple functionally related Purkinje cells, resulting in fast but coarse learning as well as significant effects on deep cerebellar nucleus and on-line motor control. In the late phase of learning decreased coupling would allow desynchronized IO firing, allowing high-fidelity transmission of error, resulting in slower but fine learning, and little on-line motor control effects.

## EFFICIENT CEREBELLAR LEARNING AND CONTROL DESPITE SPORADIC INFERIOR OLIVE SPIKING

The inferior olive (IO) neurons, via the terminal portions of the axons called the climbing fibers, form a powerful input to the Purkinje cells of the cerebellar cortex (Szentágothai and Rajkovitz, 1959; Eccles et al., 1966; Desclin, 1974). Each Purkinje cell is innervated by only a single climbing fiber from which it receives hundreds of synapses (Llinas et al., 1969; Silver et al., 1998) that collectively provide such strong excitation that their action always triggers what is referred to as a “complex spike.” Complex spikes are relatively sporadic, however, with a mean single cell firing rate of one to two spikes per second in awake animals, e.g., (Thach, 1968). Purkinje cells, which form the sole output of the cerebellar cortex, act primarily by inhibiting the deep cerebellar nuclear (DCN) neurons. The mossy fibers, via granule cells, provide the second excitatory input to Purkinje cells. In contrast to the massive input it receives from its one climbing fiber afferent, each Purkinje cell makes at most a few synapses with each of the ~100,000–200,000 parallel fibers with which it synapses (Harvey and Napper, 1991). Thus, in contrast to the all-or-none nature of the climbing fiber input, the parallel fibers provide a graded input that helps modulate the Purkinje cell “simple spike” firing rate over a range that can span 0–200 Hz.

According to the motor learning theory of the cerebellum (Marr, 1969; Albus, 1971; Ito, 1982), these two classes of Purkinje cell inputs are those that are required by a supervised learning machine, i.e., a machine that learns to improve its performance by minimizing errors (Wolpert and Miall, 1996). Specifically, Purkinje cells learn the weighting of granule cell inputs to minimize the error signals conveyed by climbing fibers (Gilbert and Thach, 1977; Kitazawa et al., 1998), via plasticity in Purkinje cell synapses (Ito, 2001). In this way, the cerebellum can learn inverse models to refine motor commands from desired states (Kawato and Gomi, 1992a; Shidara et al., 1993; Schweighofer, 1998) or forward models to predict the consequences of movements from motor commands (Kawato et al., 1987; Miall et al., 2007; Tseng et al., 2007), or both.

The cerebellum has many features that appear to match those found in effective and efficient artificial learning machines that can learn inverse or forward models from errors, such as the cerebellar model articulation controller (CMAC, Albus, 1975). Like the cerebellum, these “supervised learning” machines recode multiple inputs into high dimensional patterns (the mossy fibers to granule projections), have modifiable synapses from the high dimension layer to the output (the granule cells to Purkinje cells synapses), and use a learning rule to minimize the errors in outputs (error signals carried via climbing fibers). Differences exist, however. One crucial difference that is bound to affect the learning performance of the cerebellum negatively is that the firing rates of IO neurons are very low, which implies that a single Purkinje cell will fire at most one or two complex spikes during a typical movement. Such low firing rates significantly decrease the error transmission rate capability of the system, and thus its learning efficiency, compared to an artificial machine that is capable of high frequency transmission of errors.

To overcome this poor error transmission efficiency, the IO firing rate cannot simply be increased while maintaining good functioning of the cerebellum. One reason is that climbing fiber inputs are carried downstream by the Purkinje cells in the form of complex spikes. Assuming, for the sake of argument, that simple spikes are the only relevant output of the Purkinje cell, increases in complex spike firing rates would decrease the signal to noise ratio in the Purkinje cell output, interfering with the information being conveyed by simple spikes. In contrast, in artificial machines, because the error signal is only propagated to the level at which it is used to cause synaptic plasticity, and is not carried further downstream, it can carry any high frequency signals needed to minimize errors.

In addition to its role in motor learning, the olivo-cerebellar system may contribute directly to the on going motor commands issued by the cerebellum, and if so, complex spikes are then not simply noise, but a signal that likely needs to be distinguished from simple spikes. A long-standing argument for olivo-cerebellar activity contributing to motor commands directly is that abnormal complex spike activity patterns or lesions of the IO can cause problems in motor coordination and tremors (de Montigny and Lamarre, 1973; Llinas et al., 1975). For example, harmaline intoxication causes a tremor that is phase-locked to the highly synchronized olivo-cerebellar activity (Lamarre and Mercier, 1971; de Montigny and Lamarre, 1973; Llinas and Volkind, 1973). Olivo-cerebellar activity itself directly drives the cerebellar output that causes the tremor, rather than acting indirectly via modulation of simple spike activity because simple spikes are often absent when the tremor occurs (de Montigny and Lamarre, 1973). Further evidence that complex spikes are a significant part of Purkinje cell output comes from several studies that show that complex spikes can cause a significant inhibition of DCN activity, and that the strength of this inhibition is correlated with the level of synchrony (Bengtsson et al., 2011; Blenkinsop and Lang, 2011).

In addition, evidence exists for a direct contribution of olivo-cerebellar activity to cerebellar output under more physiological conditions. Changes in complex spike activity are associated with performance of well-learned movements. In particular, multielectrode recordings have shown that increases in complex spike synchrony levels occur in relation to conditioned tongue licking movements (Welsh et al., 1995). Subsequent imaging studies have also found that complex spike synchrony increases during motor acts (Mukamel et al., 2009; Ozden et al., 2009). However, it is important to note that the definitions of synchrony used in the imaging studies was generally more relaxed, by an order of magnitude or more, than that used in the multielectrode experiments where typically a 1 ms definition has been used (i.e., the onset of two spikes must occur within 1 ms of each other to be considered synchronous). In contrast, a variety of time bins generally ranging from 20 to 256 ms were used in the imaging studies (Mukamel et al., 2009; Ozden et al., 2009; Schultz et al., 2009). As a result, imaging studies have reported higher absolute synchrony levels than the electrophysiological studies; however, this difference is likely apparent rather than real, as it disappears when a similar temporal definition for synchrony is used and other experimental factors are accounted for; see (Lang, 2009). In sum, both electrophysiological and imaging results are consistent with a direct role for olivo-cerebellar activity in motor coordination. Yet, just as was the case with motor learning, the low firing rates of complex spikes presents a problem for the direct participation of the olivo-cerebellar system in motor coordination. Specifically, that any Purkinje cell will, on average, only fire a single complex spike during a typical movement puts severe restrictions on the ability of the olivo-cerebellar system to code motor signals in terms of individual cell firing rates.

How can the olivo-cerebellar system solve the problem of contributing both to on-line motor control and to motor learning given the constraint of low firing rate? Moreover, how can the system perform its two proposed functions independent of each other, if needed? Here, we propose that the ability of this system to modulate the level of synchronization is central to answering these questions. We address this issue as follows. First, we review the anatomical and physiological organization of the olivo-cerebellar system, with emphasis on the electrical coupling between IO cells via gap junctions. Second, we discuss how moderate electrical coupling in the IO network can, somewhat counter-intuitively, desynchronize the activity of IO neurons, and as a result, influence the learning of fine motor commands without causing unwanted motor acts. Third, we concentrate on the possible function of the closed triangle circuit formed by the IO-Purkinje cell-DCN, in the dynamic modulation of the coupling strength between IO neurons, and suggest how this circuit can modulate the transmission of errors at different stages of learning. Finally, in the discussion, we speculate how a partial dissociation of the two roles of the Purkinje cell-DCN-IO circuit in both learning and control can be made possible by their differential dependence on synchrony levels, which are controlled by feedback from the cerebellum.

## INFERIOR OLIVE NETWORK ANATOMY AND PHYSIOLOGY

The anatomical organization of the IO has two distinctive features. The first distinctive feature is that almost all (~97%) IO neurons are projection cells (Fredette and Mugnaini, 1991) whose axons do not normally give off recurrent collaterals (see De Zeeuw et al., 1996 for a discussion of this issue). As a result, few of the chemical synaptic terminals within the IO arise from the IO neurons themselves. Instead, they originate from a variety of extrinsic sources. The majority can be grouped into two classes based on their origin and chemical nature: inhibitory, gamma-aminobutyric acid (GABA)ergic synapses arise from the DCN (for most IO regions) and a few other brainstem nuclei, and excitatory synapses, which arise from a variety of brainstem and spinal cord regions (de Zeeuw et al., 1989; Nelson and Mugnaini, 1989; Fredette and Mugnaini, 1991). The second distinctive feature is that IO neurons likely form the strongest gap junction coupled neuronal network in the adult human brain (De Zeeuw et al., 1995; Condorelli et al., 1998; Belluardo et al., 2000). Thus, because direct chemical synaptic interactions between IO neurons are limited, IO neurons interact strongly via electrical synapses. Indeed, electrical coupling between IO neurons and its dependence on gap junctions has been well-established (Llinas et al., 1974; Llinás and Yarom, 1981a; Long et al., 2001; Devor and Yarom, 2002; Leznik and Llinas, 2005).

The gap junctions mainly occur between the dendritic spines of neighboring IO neurons that form the core of a complex synaptic structure known as a glomerulus (Sotelo et al., 1974). Each glomerulus, in addition to its dendritic core, contains presynaptic terminals, which can control the efficacy of the electrical coupling between specific IO neurons by a current shunting mechanism (Llinas et al., 1974; Sotelo et al., 1974; Onizuka et al., 2013). Both GABAergic and non-GABAergic terminals are found within the glomeruli (de Zeeuw et al., 1989), indicating roles for both inhibitory and excitatory control over the effective coupling of specific IO neurons. In addition to intraglomerular synapses, excitatory and inhibitory synapses occur directly on the dendrites and somata of IO neurons (Sotelo et al., 1974; de Zeeuw et al., 1989), and thus likely exert a more global control over the excitability of each IO neuron. In sum, the activity of IO neurons is modulated by excitatory inputs (such as those carrying errors), gap junctions between other IO neurons, and inhibitory inputs from cerebellar nuclear neurons (Lang, 2003).

Electrical coupling of IO neurons and modulation of its efficacy is thought to underlie the patterns of synchronous complex spike activity that are observed in cerebellar Purkinje cells (Bell and Kawasaki, 1972; Sasaki et al., 1989; Lang et al., 1999). Before discussing this relationship, however, it is worth distinguishing electrical coupling of IO neurons from Purkinje cell complex spike synchrony, because even though the latter is often used as a measure of the former, and although the two phenomena are highly related, they are not identical. Electrical coupling refers simply to there being an electrical conductance between two neurons, and its strength may be measured by a coupling coefficient (e.g., see Devor and Yarom, 2002). In contrast, Purkinje cell complex spike synchrony reflects only the synchronized suprathreshold activity between two IO neurons, and will depend on both the strength of the coupling between the two IO cells and their membrane potentials relative to spike threshold. Thus, for example, if one of two coupled cells is more hyperpolarized, it may not fire an action potential, even when excited by current flowing from the other cell, and thus the complex spike activity in the Purkinje cells postsynaptic to these neurons will not be synchronized. Indeed, such a scenario has been postulated to explain some of the changes in synchrony distribution that occur following block of excitatory drive to the IO (Lang, 2001, 2002). Nevertheless, in most instances the level of complex spike synchrony is probably a good indicator of electrical coupling between IO neurons.

The patterns of synchronous complex spike activity that characterize the olivo-cerebellar system have been investigated *in vivo* during the past several decades using multielectrode recording. Consistent with the gap junction coupling of IO neurons underlying complex spike synchrony, both synchronous IO and complex spike activity is lost when IO gap junctions are blocked pharmacologically (Leznik and Llinas, 2005; Blenkinsop and Lang, 2006), and is absent in connexin36 knockout mice (Long et al., 2001; Marshall et al., 2007). Furthermore, gap junctions, together with cellular current dynamics, generate synchronized subthreshold oscillations in the membrane potential of IO neurons (Llinás and Yarom, 1981a, b; Manor et al., 1997; Schweighofer et al., 1999).

These studies showed that the spatial distribution of synchronous complex spike activity is rather restricted despite the extensive gap junction coupling of IO neurons. Complex spike synchrony can occur between specific widely separated regions of the cortex (De Zeeuw et al., 1996); however, the highest levels of synchronous activity are found mainly among Purkinje cells located in the same narrow (~250–500 μm wide) cortical band, with the long axis of each band oriented parallel to the transverse axis of the folium in which it is located (Sasaki et al., 1989; Sugihara et al., 1993; Lang et al., 1999). Although the spatial resolution of most of these studies was only ~250 μm (the spacing of the electrodes in the array), there is good reason to believe that finer grained patterns than the observed banding patterns are unlikely to exist, because recording with higher density multielectrode arrays (166 μm electrode spacing) failed to reveal any finer intraband structure (Fukuda et al., 2001), nor did studies with calcium imaging techniques, which, in theory, can record complex spikes from the entire local Purkinje cell population albeit with less temporal resolution (Mukamel et al., 2009; Ozden et al., 2009; Schultz et al., 2009). The synchrony bands are at least partly congruent with anatomically-defined compartments based on zebrin staining, as high synchrony levels are found mainly among cells within the same zebrin compartment (Sugihara et al., 2007), and thus reflect the topography of the olivo-cerebellar projection (Voogd and Bigaré, 1980).

However, complex spike synchrony is a dynamic entity as shown by changes in complex spike synchrony levels and patterns associated with movement (Welsh et al., 1995; Mukamel et al., 2009; Ozden et al., 2009). The control of the specific synchrony patterns reflects the activity of GABAergic and glutamatergic inputs to the IO. Intra-IO injection of picrotoxin (PIX), a GABA-A antagonist, or lesion of the GABAergic projection from the cerebellar nuclei, induces higher complex spike firing rates, and more widespread synchronization (Lang et al., 1996; Lang, 2002). Consistent with these *in vivo* findings, voltage-sensitive dye imaging results have demonstrated that PIX increases the size of coherently oscillating IO neuronal clusters in brainstem slice preparations (Lang et al., 1997). In contrast to blocking GABA, blocking glutamatergic activity produces lower firing rates and smaller, more discrete groups of Purkinje cells with synchronized activity (Lang, 2001, 2002).

**FIGURE 1:**
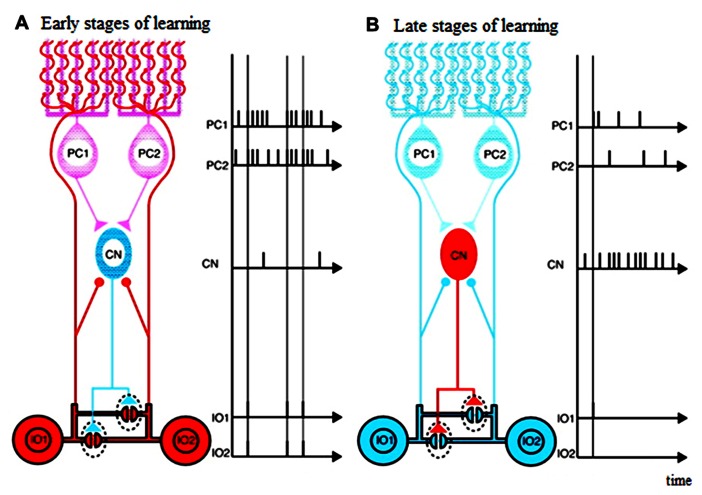
**Schematic diagram illustrating possible functions of closed triangle circuit consisting of inferior olive (IO) nucleus, Purkinje cells (PCs), and deep Cerebellar Nucleus (CN).** **(A)** Early stages of learning. **(B)** Late stages of learning. Purkinje cells inhibit deep cerebellar nucleus cells. Inhibitory deep cerebellar nucleus cells innervate dendrites of inferior olive cells within glomeruli very close to gap junctions. The diagram does not include mossy-fiber inputs and their target granule cells, parallel-fiber inputs, inhibitory interneurons, excitatory deep cerebellar nucleus. Excitatory inputs to inferior olive cells are not shown either. Blue neurons are not excited, and red are excited. Excitatory synapses are shown by circles, and inhibitory synapses are shown by triangles. Horizontal lines show electrical gap junctions. Adapted from Figure 3 in Kawato et al. (2011), itself adapted from Figure 3 in De Zeeuw et al. (1998)

That the GABAergic afferents to the IO largely arise from the DCN suggests that the cerebellum actively shapes its own inputs. Indeed, the topography of the connections between the IO and cerebellum allow functionally related Purkinje cells, DCN cells, and IO cells to be grouped into “microcomplexes” or modules (Ito, 1984; Schweighofer, 1998; Apps and Hawkes, 2009). That is, the connections between the IO and cerebellum are precisely aligned so that anatomically closed loops are formed between corresponding regions of the IO, cerebellar cortex and nuclei (Voogd and Bigaré, 1980; Sugihara and Shinoda, 2004; Apps and Hawkes, 2009; Sugihara et al., 2009; Ruigrok, 2010). Thus, the cerebellar cortex can be subdivided into numerous longitudinal zones, and Purkinje cells from anyone zone will target a specific region of the cerebellar (or in a few cases, the vestibular) nuclei, exerting an inhibitory influence on those neurons. In turn, about 30–50% of the cerebellar nuclear neurons from each regions end inhibitory projections to a particular IO region (de Zeeuw et al., 1989; Nelson and Mugnaini, 1989; Fredette and Mugnaini, 1991). Thus, a double- inhibitory feed back circuit from Purkinje cells to the IO via the DCN exists, and enables each cerebellar cortical region to influence the activity of its own projection from the IO (see **Figure 1**). Consistent with this anatomical arrangement, complex spike synchrony bands appear to follow this modular organization (Sugihara et al., 2007), and the simple spike activity of each cortical region, via this feedback circuit, can regulate its own complex spike synchrony levels (Marshall and Lang, 2009).

In sum, it seems clear that synchrony is likely to be a physiologically important parameter of olivo-cerebellar function. However, there is less consensus on what its function or functions may be. This lack of consensus is certainly due to several factors, but in the following we will focus on the problems related to motor learning and specifically how electrical coupling of IO neurons can allow learning processes to occur at complex spike firing rates and synchrony levels that do not interfere with on-line motor coordination.

## CHAOTIC RESONANCE ENHANCES LEARNING BY INCREASING INFORMATION TRANSMISSION

### INTERMEDIATE COUPLING LEADS TO CHAOS AND INCREASE INFORMATIONTRANSMISSION VIA “CHAOTIC RESONANCE”

Coupled IO cells do not necessarily synchronize their firing, and indeed, although the system has the capability of generating widespread synchrony, it normally does not do so. Several results suggest that electrical coupling among IO neurons may allow other patterns of activity. First, in coupled oscillatory cell IO models, depending on coupling strength, the neurons can fire in phase or antiphase (Schweighofer et al., 1999). Antiphase firing of IO neurons has, in fact, been observed experimentally under *in vitro* conditions (Llinás and Yarom, 1986). Second, in networks of IO cells, coupling can induce chaos in subthreshold oscillations (Makarenko and Llinas, 1998). Moreover, in a model of IO neurons, a maximum chaotic regime of spiking activity was observed for intermediate levels of gap junction conductance (Schweighofer et al., 2004), whereas lower and higher coupling strengths induced more regular firing. Note that chaotic systems are deterministic, which means that their future behavior is fully determined by their initial conditions, with no randomness (noise) involved. However, small differences in initial conditions yield widely diverging outcomes, rendering long-term prediction impossible.

Our previous modeling study (Schweighofer et al., 2004) also indicated that such chaotic behavior can enhance information transfer in these neurons, via a “chaotic resonance” (Nishimura et al., 2000). We quantified the influence of electrical coupling on the low firing code of IO output by computing how much information about the input could be extracted from the IO output spike trains, i.e., the mutual information. The concept of chaotic resonance derives from that of stochastic resonance (see Wiesenfeld and Moss, 1995 for review), a phenomenon in which the presence of noise helps a non-linear system in amplifying a weak (under threshold) signal, as found in sensory neurons. Considering that deterministic chaos resembles the feature of noise and provides a source of fluctuation, stochastic resonance-like behavior can be observed in deterministic dynamical systems in the absence of noise in two ways: either by substituting the stochastic noise source by a chaotic source, or directly via intrinsic chaotic dynamics, as we found in IO networks with intermediate coupling strengths.

In the chaotic regime of IO network, we have shown that the increase in information transmission in IO neurons is achieved via distributing-frequency components of the error inputs over the sporadic, irregular, and non-phase-locked spikes (Schweighofer et al., 2004). Desynchronization is indeed necessary for scattering the spike timings of each neuron to increase the time resolution of the population rate coding (Masuda and Aihara, 2002). Then, the complete continuous error signal can be reconstructed by spatial integration across Purkinje cells within a microcomplex and via temporal integration for each Purkinje cell via cumulative effects of long-term depression (LTD; see Figure 1 and associated text in Schweighofer et al., 2004 for an intuitive understanding).

Note that these results are robust to cell parameters and complexity and do not depend on the specificity of the cell model. In our original chaotic IO model, we used a rather complicated compartment model, and many physiological parameters were chosen rather arbitrarily, casting doubt on the generalizability of our results. In Tokuda et al. (2010), however, we showed that a simple, minimal, model of IO neurons also exhibits chaotic resonance for intermediate coupling.

### INCREASED INFORMATION TRANSMISSION LEADS TO MORE EFFICIENT LEARNING

In Tokuda et al. (2010), we tested the prediction that efficient cerebellar learning is realized with an intermediate coupling strength. In these simulations, the IO neurons provide error signals to an idealized model of the cerebellar cortex that learns, via feedback error learning (Kawato et al., 1987; Kawato and Gomi, 1992b) to control a simplified model of the human arm in rapid reaching movements. As predicted, intermediate coupling levels, which allow chaotic resonance and increased information transfer of the error signals, accelerated motor learning models, despite the low IO firing rate (Tokuda et al., 2010).

Note that noise, ubiquitous in the nervous system, can have a similar effect to coupling in enhancing cerebellar learning via stochastic resonance (Tokuda et al., 2010). Indeed, we showed that noise and coupling are complementary and reinforce each other: the interplay between coupling and noise enlarged the parameter ranges of both coupling strength and noise intensity that provide efficient learning. However, chaos-induced desynchronization, possibly in addition to noise-induced desynchronization, is advantageous in two ways. First, from an energetics point of view, coupling generated chaos is a cheaper way of destroying the synchrony between cells, because noise in the nervous system is thought to arise mainly from synaptic noise (Hubbard et al., 1967). On the other hand, electrical coupling itself does not require energy expenditure. Second, although coupling could be modulated during learning by inhibitory inputs from the cerebellar nucleus, it is unclear how noise could be modulated during learning.

Experimental support for this role of electric coupling in cerebellar learning comes from mice mutants lacking electrotonic coupling between IO cells (Van Der Giessen et al., 2008). These mice have no prominent general motor deficits, but they do exhibit deficits in learning-dependent motor tasks such as locomotor or eye-blink conditioning. The IO neurons in these mice have altered subthreshold oscillations, resulting in more variable latencies of spikes, which lead to deficits in the timing of conditioned motor responses (Van Der Giessen et al., 2008). Similarly, humans with reduced IO coupling as a result of the anti-malaria drug mefloquine exhibit no general motor deficits but show motor learning impairments (van Essen et al., 2010).

## DYNAMIC MODULATION OF IO ELECTRICAL COUPLING DURING LEARNING

### MODULATION OF COUPLING VIA INHIBITION FROM NUCLEAR CELLS

In recent simulation work, we investigated whether inhibitory modulation of electrical coupling is indeed a major determinant of the IO firing dynamics (Onizuka et al., 2013). We specifically aimed at reproducing the IO firing dynamics of the PIX and carbenoxolone (CBX) experimental studies (Lang, 2002; Blenkinsop and Lang, 2006). The original model by Schweighofer et al. (1999) was modified by adding a model of the glomerulus comprised of dendritic spine necks that accommodate gap junctions and inhibitory synapses (see **Figure 1**).In this model, under simplifying assumptions, the effective coupling conductance *g*_effective_ between connected IO cells is computed from the gap junction conductance *g*_junction_ and the conductance of inhibitory synapses *g*_inhibitory_ and from the spine neck conductance *g*_spine_ as follows (Katori et al., 2010):

$$g_{\text{effective}}=(g_{junction}\cdot g_{spine})/\left({2g}_{junction}+g_{spine}+g_{inhibitory}\right)$$

Thus, if the inhibitory synaptic conductance is large, the effective coupling conductance decreases because of shunting inhibition. In (Onizuka et al., 2013), we determined the gap junction conductance *g*_j__u__nctio__n_ and the conductance of inhibitory synapses *g*_i__n__hibit__o__r__y_ that minimize the fitting error between simulated IO firing from the model and the experimental complex spike data in three conditions: PIC, CBX, and control. We found that the inhibitory *g*_i__n__hibit__o__r__y_ and gap junction *g*_j__u__nctio__n_ conductances roughly halved under the PIX and CBX conditions, respectively, supporting the role of a direct modulation of coupling strength via inhibitory inputs. Thus, because the inhibitory neurons controlling the strength of coupling between IO cells are located in the DCN, the strength of effective coupling, and thus the level of chaotic behavior, presumably depends on the modulation of the deep cerebellar neurons via plastic processes in the cerebellar cortex and nuclei.

Experimental support for a functional role of the inhibition near gap junctions was previously reported (Shaikh et al., 2010). It was argued that oculopalatal tremor may be due to the removal of inhibition near the electronic gap junctions in the IO. Interestingly, such patients with oculopalatal tremor show slower motor learning. This could be explained by the fact that only poorer error information can be transmitted when IO cells are strongly coupled and oscillate in-phase (Schweighofer et al., 2004).

### DYNAMIC MODULATION OF IO ELECTRICAL COUPLING VIA THE PURKINJE CELL-DEEP CEREBELLAR NEURONS – IO TRIANGLE

The Purkinje cell-DCN -IO triangle may act as a circuit to satisfy the motor learning requirements of the cerebellar learning system (Kawato et al., 2011). That is, in the early phase of motor learning, when motor acts are clumsy and far from the desired ones and the executed movement trajectories are perturbed, the motor plans and commands both need to be grossly modulated. Conversely, in the late phase of the learning, when the motor acts become skillful and the movement trajectories are smooth and close to the desired ones, the motor plans and commands need only fine tuning.

The mosaic structures of the cerebellar system where the IO-Purkinje cell-DCN loop is topographically organized in “microcomplexes” may help such modulation of motor learning. The neural events to meet these motor learning requirements would be massive climbing-and mossy-fiber inputs to the Purkinje cells in the early phase of motor learning (leading to low DCN activity), and small mossy-and climbing- fiber inputs in the late phase. In the early phase of learning, highly effective coupling across the IO neurons due to low DCN activity would allow widespread synchronized IO firing in response to error signals, which could potentially lead to synaptic weight changes in many Purkinje cells. Cerebellar learning would be fast but coarse. Conversely, in the late phase of learning, if IO neuronal firing becomes less synchronized, synaptic changes would occur among more restricted Purkinje cell groups, which would allow more subtle modifications in the final learning stages (compare left and right panels in **Figure 1**).

In Tokuda et al. (2012), we conducted simulations to examine the advantage of the adaptive coupling strength over fixed coupling strength during motor learning. IO neurons transmitted error signals in a feedback-error learning scheme to learn the inverse dynamics of a two-dimensional arm. In the adaptive coupling condition, the coupling strength between the IO neurons was slowly decreased as learning proceeded. The error signals amplitudes were large early in learning because movements were mainly under feedback control. Feedback control in biological motor control is slow and inaccurate because of the low feedback gains necessary to avoid oscillations and divergence due the long feedback delays; see for instance (Schweighofer et al., 1998). As learning of the internal inverse model proceeded, the movements became straighter and the error signals became smaller. Since the small error signals provided only a weak influence on the IO neurons, weak coupling was needed to maintain the desynchronized neural activities. Results showed that adaptive coupling led to a more efficient learning process than with a fixed coupling strength.

## DISCUSSION

We have reviewed experimental and computer simulations studies suggesting that the Purkinje cell-DCN -IO circuit may act as a self-regulating circuit that potentially has two functional roles, one in motor learning and one in on-line motor control. That is, the control of synchrony between IO complex spikes via modulation of electrical coupling could enhance cerebellar learning and on-line motor control. If the olivo-cerebellar system has two functional roles, then ideally it would be best if the performance of each function was controlled independently. Here, we suggest that modulation of the effective electrical coupling of IO neurons, and there by the levels of complex spike synchrony, may allow at least semi-independent control.

Specifically, the olivo-cerebellar system could contribute to motor commands primarily when it is operating in a relatively synchronized state. Synchronization, coupled with the convergence of the Purkinje cell to DCN pathway, would allow complex spikes to be distinguished from ongoing simple spike activity, and therefore they could alter the activity of DCN neurons in distinct ways from the latter signals. Thus, synchronous complex discharges would make a contribution to outgoing cerebellar motor commands distinct from that made by simple spike activity. In contrast, when complex spike activity is desynchronized it may not contribute significantly to motor commands, because in this state complex spikes may be less distinguishable from simple spike activity. It is possible that the burst nature of the complex spike may still allow the DCN neurons to distinguish them from simple spikes; however, the extent to which the secondary spikes of each complex spike are propagated is debated (Ito and Simpson, 1971; Campbell and Hesslow, 1986; Khaliq and Raman, 2005; Monsivais et al., 2005). Nevertheless, based simply on the firing rate superiority of simple spikes, it seems plausible that asynchronous complex spike activity would generally make a less significant direct contribution to shaping DCN activity. The olivo-cerebellar system may switch into an on-line motor control state for two causes, internal or external with respect to the cerebellum. On one hand, high synchrony states may be due to increased effective coupling levels among IO cells via low activity in the subset of DCN neurons that project to the IO. In this case, simple spikes would, via their action on the DCN, help in determining whether or not olivo-cerebellar activity will contribute to the upcoming motor command. On the other hand, large, highly synchronized volleys in excitatory afferent IO pathways could lead to synchronous complex spikes, which could also trigger a motor response.

In contrast to motor control, the potential for triggering motor learning exists regardless of synchrony level, because the ability of complex spikes to modulate synaptic plasticity at a single cell level are not affected by synchrony levels: plastic processes intrinsic to any one Purkinje cell caused by its firing a complex spike would be expected to be independent of the number of other Purkinje cells generating complex spikes at the same time (but see paragraph below). The overall speed of motor learning would be modulated by synchrony levels, however. In the early phases of learning, initially strong coupling would allow transmission of large errors to multiple functionally related Purkinje cells, resulting in fast but coarse learning (in addition to its significant effects on deep cerebellar nucleus and on-line motor control). In contrast, in the late phase of learning, decreased coupling would lead to desynchronized IO firing, allowing high-fidelity transmission of error, resulting in slower but fine learning, and little on-line motor control effects. We proposed that the desynchronized state arises via higher inputs from DCN activity. Thus, by modulating the effective coupling among IO cells, the DCN may control the characteristics of error signals sent to the cerebellum, once again acting in a self-regulating manner. Our proposal is at least in part coherent with data from (Milak et al., 1995) showing that the activity of DCN neurons increase above background activity during motor learning. However, our proposal, in its current form, does not account for the additional results of Milak et al. (1995) showing that DCN activity progressively decreased as the task became well practiced. Perhaps excitatory and inhibitory inputs from the DCN to the IO control cellular activity and coupling in a non-linear manner, as suggested by our model of IO neuron, which is only firing for a limited range of inputs (see Figure 4 in Schweighofer et al., 1999). Additional work is needed to shed light on the effect of excitatory inputs on IO activity and coupling.

In addition to synchronized IO activity in the early phase of learning, learning can further be accelerated by IO neurons firing in bursts (Eccles et al., 1966; Crill and Kennedy, 1967). These bursts potentially allow the IO neurons to communicate in a more refined way than just binary, thereby increasing bandwidth (Maruta et al., 2007; Bazzigaluppi et al., 2012). The number of spikelets in a burst has been linked to the strength and type of long-term plasticity induced by climbing fiber activation (Mathy et al., 2009). Thus if synchronization of IO neuronal activity affected spikelet number, synchrony would be another possible mechanism by which the olivo-cerebellar system regulates learning processes in the cortex. However, the exact relationship of synchrony and spikelet number needs further study. The amplitude of subthreshold oscillations in IO neurons is an alleged surrogate for synchronization level of IO neuronal activity and simulations of IO networks, and *in vivo* recordings suggest that an inverse relationship exists between the amplitude of the subthreshold oscillation and IO spikelet number (Bazzigaluppi et al., 2012; De Gruijl et al., 2012). Thus, modulation of spikelet number is an intriguing possible mechanism for enhancing the control that the olivo-cerebellar system exerts over Purkinje cell plasticity, and in the role it plays in shaping motor commands sent to the DCN.

In any case, the above implies that by limiting synchrony levels, feedback from the cerebellum would enable the olivo-cerebellar system to allow modification of synaptic weights without causing movements. However, the separation of function is not complete, because synchronous complex spike activity would, in the currently proposed scheme, cause both generation of movements and synaptic plasticity. This implies that each time complex spikes contribute to movement generation, the circuitry generating the movement is altered, and thus the mapping of brain activity to movement is modified. This is in some ways analogs to the proposal that the process of memory retrieval may modify the memory trace itself (Sara, 2000). Indeed, it may partly explain the fact that even in highly skilled athletes and musicians the performance of highly practiced motor acts still retains some variability (e.g., as of 2013, the highest free throw percentage for a season by a player in the National Basketball Association is only 90.4% http://www.nba.com/statistics/default_all_time_leaders/AllTimeLeadersFTPQuery.html?top). Conversely, subtle modification of cerebellar circuits could underlie the efficacy of taking practice swings before hitting in baseball or similar warm up routines.

Finally, it is worth considering, in the context of the motor learning process, cases where truly high synchrony levels may occur. In the early phase of motor learning the motor plans and commands both need to be grossly modulated. Motor acts are clumsy and far from the desired ones and the executed movement trajectories are likely to be perturbed as a result. Consistent with this hypothesis, in motor learning of arm reaching under novel force fields, changes in motor commands are large for the first few trials, much more than the level of trajectory errors (Franklin et al., 2008). Such perturbations, if they resulted in a synchronous afferent volley to the IO, would be away to elicit widespread synchronous complex spike activity, and thus possibly elicit corrective movements, and perhaps more importantly to allow large-scale changes in synaptic connectivity. As the learning process continues, the motor acts become skillful and the movement trajectories become smooth and close to the desired ones. In this case, there is less likely to be major perturbations with highly synchronized complex spike activity resulting. Instead, motor plans and commands need only fine-tuning and the olivo-cerebellar system may generate relatively desynchronized activity that would drive such fine-tuning.

## Conflict of Interest Statement

The authors declare that the research was conducted in the absence of any commercial or financial relationships that could be construed as a potential conflict of interest.
